# Evidence for post-transcriptional regulation of clustered microRNAs in Drosophila

**DOI:** 10.1186/1471-2164-12-371

**Published:** 2011-07-19

**Authors:** Sergei S Ryazansky, Vladimir A Gvozdev, Eugene Berezikov

**Affiliations:** 1 Institute of Molecular Genetics, Russian Academy of Sciences, Moscow, Russia; 2 Hubrecht Institute, Royal Netherlands Academy of Arts and Sciences and University Medical Centre Utrecht, Utrecht, The Netherlands; 3 Institute of Cytology and Genetics, Russian Academy of Sciences, Novosibirsk, Russia

## Abstract

**Background:**

MicroRNAs (miRNA) are short 21-23nt RNAs capable of inhibiting translation of complementary target messenger RNAs. Almost half of *D. melanogaster *miRNA genes are grouped in genomic clusters.

**Results:**

The peculiarities of the expression of clustered miRNAs were studied using publicly available libraries of sequenced small RNAs from different Drosophila tissues. We have shown that although miRNAs from almost all clusters have similar tissue expression profiles (coordinated clusters), some clusters contain miRNAs with uncoordinated expression profiles. The predicted transcription start sites (TSSs) of such clusters are located upstream of the first miRNA, but no TSSs are found within the clusters. The expression profiles of miR and miR* sequences in uncoordinated clustered miRNAs do not correlate while their profiles from the coordinated clustered miRNAs are similar.

**Conclusions:**

The presence of exclusively upstream promoters in miRNA clusters containing uncoordinated miRNAs means that the clusters are transcribed as single transcription units. The difference of tissue expression profiles of uncoordinated miRNAs and the corresponding miRs* suggests a post-transcriptional regulation of their processing or stability.

## Background

MicroRNAs (miRNAs) are short 21-23 nt non-coding RNAs that are processed from one of the arms of hairpin-like 60-100 nt precursor miRNAs (pre-miRNAs). Pre-miRNAs are produced from primary pri-miRNAs transcribed from miRNA genes by RNA polymerase II. Mature miRNAs (miRs) trigger posttranscriptional regulation of the target mRNAs mediated by a specific set of effector proteins [1]. The perfectly complementary miRs induce mRNA degradation in plants, while in animals the partially complementary miRs cause mainly mRNA degradation but also the blocking of translation [2-4]. MiRNA-mediated inhibition of target mRNA translation is considered to be a powerful mechanism of gene expression regulation. Besides the prevalent mature miRs, the minor star molecules (miR*) are generated from the opposite arm of the pre-miRNAs and sometimes also capable to inhibit expression of target mRNAs [5,6]. The choice of pre-miRNA strand producing the mature miR is determined by the strands' sequences.

Up to several hundred of miRNA genes are present in eukaryotic genomes, and often miRNAs are located close to each other in a genome, forming genomic clusters [7,8]; for instance, *Drosophila melanogaster *has at least 176 miRNA genes (miRBase v.16) and almost half of them are clustered. Clustered miRNAs are often co-expressed [9-12] and can jointly regulate functionally related genes, e.g. included in the same signaling pathway [1,12-14]. Obviously, the regulation of the expression of individual clustered miRNAs can fine-tune the pathway modulation. Since clusters are considered to be transcribed as single primary pri-miRNA transcripts [7,8,15-17], such regulation can be achieved by post-transcriptional regulation of miRNA maturation [18-21]. In this report we found that several *Drosophila *miRNA clusters contain miRNAs with the expression profiles different from the profiles of the other miRNAs in the same cluster. Our data argue in favor of a contribution of post-transcriptional rather than transcriptional regulation to the tissue-specific expression of these uncoordinated miRNA clusters.

## Results and discussion

### Overview of miR clusters

Here we refer to the grouped miRNA genes as miRNA cluster if they are located not more than 1 kb apart. A summary of 20 Drosophila miRNA clusters is presented in Additional file 1, Table S1. Using the above criterion, dme-mir-310, -311, -312, -313, -991 and -992 should be related to a single cluster, but the analysis of pair correlation coefficients of their tissue expression profiles (see below) shows that dme-mir-310, -311, -312 and -313 and dme-mir-991, -992 are clearly separated into two clusters, as has been noted before [11]. The 281 cluster, containing a tandem of identical and indistinguishable dme-mir-281-1 and dme-mir-281-2 was excluded from the further expression analyzes. Similarly, identical dme-mir-6-1, -6-2, -6-3 from the 6~309 cluster, dme-mir-2a-1, -2a-2 from the 2a~2b cluster, and dme-mir-983-1, -983-2 from the 983~984 cluster were considered as a single miRNA within each cluster. The subsequent expression analysis showed an adequacy of this simplification. In total, we have examined 19 miRNA clusters.

### Some clustered miRNAs have uncoordinated expression profiles

**Figure 1:**
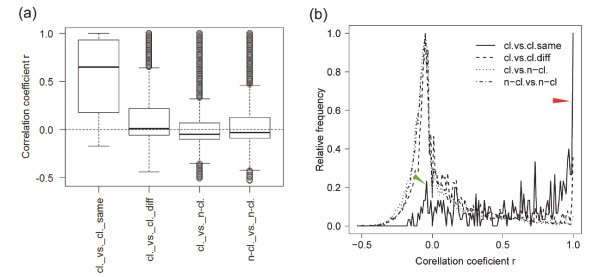
**miRNAs with similar expression profiles mainly belong to the same clusters**. **(a) **Pearson correlation coefficients *r *of expression profiles for miRNA pairs from the same cluster (cl.vs.cl.same), different clusters (cl.vs.cl.diff), clustered and non-clustered miRs (cl.vs.n-cl), and non-clustered miRNAs (n-cl.vs.n-cl.). **(b) **Frequency distributions of *r *in the same groups of miRNA pairs. Red and green arrows indicate highly and low correlated fractions of the clustered miRNAs in cl.vs.cl.same group, respectively. Distributions were normalized to the maximum value of frequency in each group.

To determine the miRNA expression profiles, we analyzed 16 million reads of sequenced small RNAs from 9 publicly available libraries prepared from heads, bodies, testes, ovaries, embryos and S2 cells (Additional file 1, Tables S2 and S3). The coordinated expression of clustered miRNAs has been described earlier in human, mouse and fruit fly [9-12]. Consistent with these data, we showed that in contrast to miRNAs from different clusters or non-clustered miRNAs, miRNAs from the same cluster tend to be co-expressed in the similar set of tissues (*P*-value < 2e-16, t.test) (Figure 1a). The evaluation of the frequency distributions of the miRNA correlation coefficients of expression profiles also demonstrates that highly correlated miRNAs are mainly related to miRNAs from the same cluster (cl.vs.cl.same, red arrow on the Figure 1b), but not to miRNAs from different clusters. Therefore, expression of miRNA clusters is regulated independently to each another.

While the expression profiles of miRNAs in 14 clusters have a high coefficient of correlation (*r *> 0.6) (Additional file 2), a small fraction of clustered miRNAs have a low correlation coefficient of the expression profiles (cl.vs.cl.same, green arrow on the Figure 1b). Indeed, inspecting the pair correlation coefficient values and heatmaps revealed that 5 clusters (283~12, 275~305, 9c~9b, 100~125, 13b-1~2c) contain miRNAs, the profiles of which differ from the rest of the miRNAs in the same cluster (*r *< 0.3) (Table 1, Additional file 2). The expression levels of uncorrelated miRNAs are sufficiently high and similar to the correlated ones (*P*-value = 0.27, t.test, Additional file 1, Table S3), thus the low correlation values of expression profiles are not due to random noise in miRNA detection. In the 13b~2c cluster a wrong conclusion of a lack of correlation of dme-mir-13b-1 to dme-mir-13a and dme-mir-2c can be formally deduced due to the superposition of the expression of the non-clustered paralog dme-mir-13b-2 with identical sequence. The other 4 clusters are likely to have genuinely uncoordinated miRNAs. For instance, the expression profiles of dme-mir-304 and dme-mir-12 in cluster 283~12 correlate to one another (*r *= 0.69), but at the same time the profile of dme-mir-283 does not correlate to both dme-mir-304 (*r *= 0.04) and dme-mir-12 (*r *= 0.02).

**Table 1:** Clusters with uncorrelated miRNAs

Cluster name	Uncorrelated miRNA^1^
100~125	dme-mir-100
13b~2c	dme-mir-13b-1
275~305	dme-mir-305
283~12	dme-mir-283
9c~9b	dme-mir-306

^1^Uncorrelated miRNAs are characterized by low correlation coefficient with the corresponding miR*; although the 100~125 cluster possibly has two uncorrelated miRNAs, dme-let-7 and dme-mir-100, only dme-mir-100 does not correlate to dme-mir-100* (see text for details).

### Promoters of miR clusters

Coordinated expression of clustered miRNAs can be explained by the transcription of a whole cluster as a single polycistronic precursor RNA driven by an upstream promoter, as has been reported earlier [15-17,22,23]. Using RNA-seq and tilling microarrays of RNA samples from 30 fly developmental stages, single pri-miRNAs for 16 non-clustered miRNAs as well as 7 miRNA clusters have been identified [23]. By contrast, uncoordination of the expression profiles of some clustered miRNAs may result from independent transcription of these miRNAs from their own promoters. Moreover, although polycistronic transcript of the uncoordinated 100~125 cluster has been detected in pupae and ecdysone-treated S2 cells [22], it cannot rule out a possibility of the presence the tissue-specific intra-cluster promoters.

**Figure 2:**
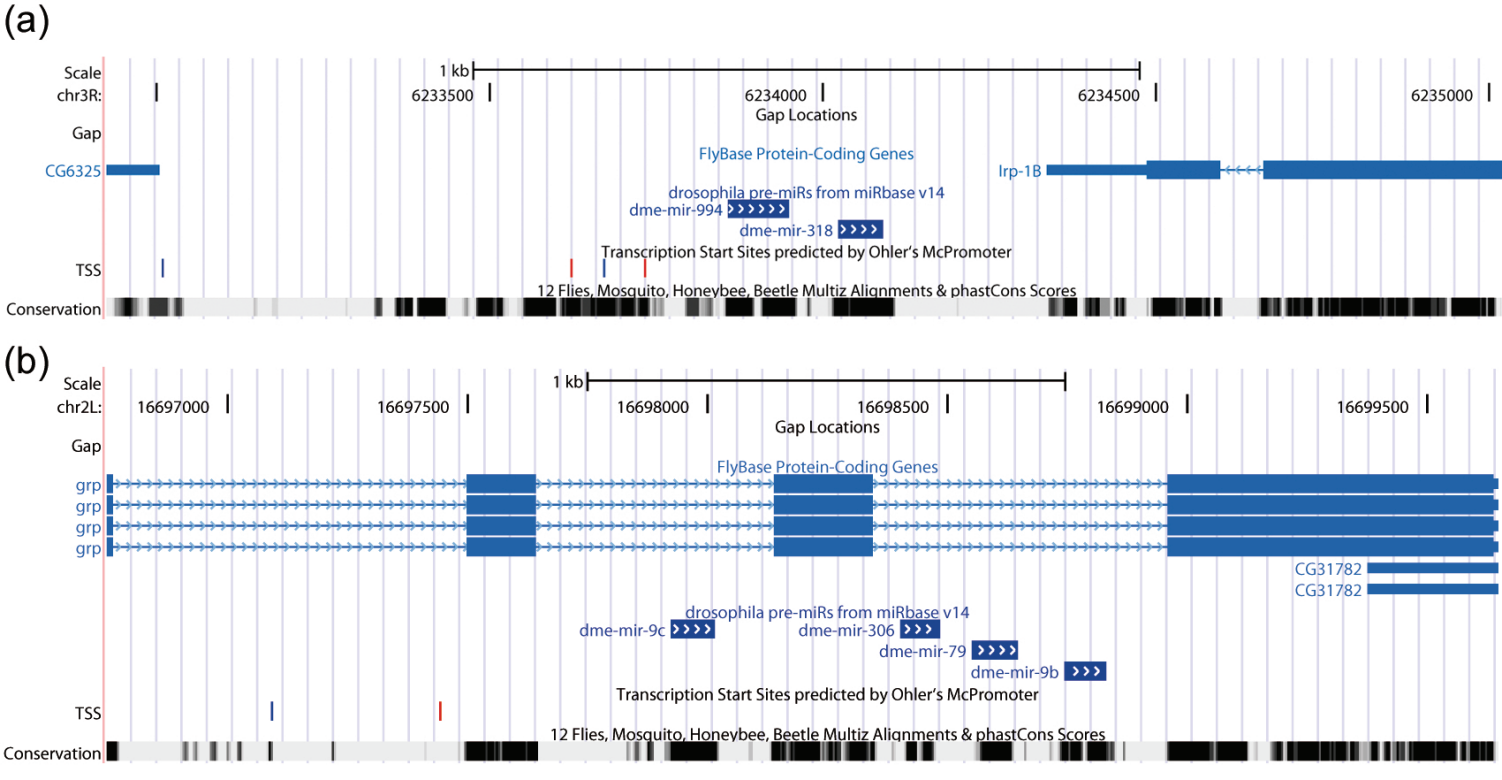
**TSSs of 994~318 and 9c~9b clusters**. **(a) **Two putative TSSs of the 994~318 cluster with coordinated expression profile. **(b) **One putative TSS of the intronic 9c~9b cluster with uncoordinated expression profile. Red and blue ticks are TSSs in sense and antisense orientations, receptively. The maps were produced in the UCSC Genome Browser [40].

To test a possibility of independent transcription of clustered miRs, we have evaluated the positions of their putative promoters *in silico*. We focused on the search of RNA polymerase II promoters because only a few miRNA genes have RNA polymerase III promoters [24]. It has been shown that transcription start sites (TSSs) of Drosophila pol II promoters associate with the specific pair combinations of sequence motifs [25-27]. In our analysis of cluster promoters we have used a set of TSSs predicted to be located near the detected combinations of such motifs *(McPromoter006*, [26]). Similar approaches of promoter finding by localization of putative TSSs for human miRNAs have been performed earlier [28,29]. Two examples of putative TSSs for coordinated and uncoordinated clusters are shown in Figure 2. The full list of cluster TSSs is presented in Additional file 1, Table S4 and Additional file 3.

The starts of 5'-ends of pri-miRNAs of coordinated 1002~968 and 277~34 clusters [23] are coincided with location of the predicted TSSs within 300 nt range. The 277~34 cluster also has intra-cluster TSSs upstream of dme-mir-34. The next 5 clusters containing miRNAs with coordinated expressions (982~303, 959~964, 994~318, 983~984, 310~313) have one or several TSSs upstream of the first miRNA (Figure 2a) but not within the clusters. Another 3 clusters (972~974, 975~977, 978~979) are neighbors and have 2 putative TSSs - the first TSS is located upstream of the 972~974 and the second one is located inside the 972~974 and upstream of the 975~977 and 978-979 clusters. This suggests that the 972~974 cluster may be transcribed from the first TSS and both TSSs may be used to ensure transcription of the other two clusters. The next two coordinated clusters, 998~11 and 2a~2b, are encoded in introns of protein-coding genes and have no predicted TSSs, and coordination of their expression can be explained by the transcription from the host genes promoters. We failed to detect any TSS for the 309~6 cluster, but its single promoter as well as precursor have been identified and experimentally verified earlier [15,23]. No nearest TSSs were found for the last 991~992 cluster. Overall, almost all correlated miRNA clusters (12 out of 14) contain only upstream putative pol II promoters ensuring transcription of the whole cluster and coordination of clustered miR expressions (Additional file 3 and Additional file 1, Table S4).

Surprisingly, similarly to the coordinated clusters, the 100~125, 275~305, 9c~9b, 283~12 and 13b~2c clusters containing miRNAs with uncoordinated expression patterns, also have one or several TSSs only upstream of the first miRNA in a cluster (Figure 2b). None of these 5 clusters have any TSS near the internal miRNAs (Additional file 1, Table S4 and Additional file 3).

The distances between miRNAs within clusters usually small (mean 213 nt, median 75 nt), and a lack of putative TSSs can be explained by a low probability of finding them there. To estimate this probability, we calculated an empirical cumulative distribution function (ecdf) of lengths from the starts of fly miRNAs to the nearest upstream putative TSS. Then, the probability *P *to observe a TSS in a region of the given length *x *can be determined as *P *= ecdf(*x*). Indeed, the short inter-miRNA spaces seem to explain the absence of TSSs within miRNA clusters since their mean *P *= 0.1 and median *P *= 0 (Additional file 1, Table S5). There are also five 500-900 nt regions with probability 0.33≤P ≤ 0.44 from coordinated and uncoordinated clusters, one and only one of which (from coordinated 277~34 cluster) contains two TSSs, and this is fully consistent with statistical prediction.

Moreover, to estimate the accuracy of *McPromoter *in the prediction of miRNA TSSs we checked an enrichment of the TSSs by pol II using the modENCODE ChIP-seq data. We have found that all putative TSSs of 9 miRNA clusters are located in pol II enrichment regions, demonstrating that *McPromoter *have a very high specificity in TSSs prediction (Additional file 3). The location TSSs of the other clusters near the pol II enriched exons of genes prevents their precise attribution to TSSs. Except for a 277~34, none of the large cluster intra-miRNA regions are enriched by pol II. In addition, there are no significantly conservative regions upstream of uncorrelated miRNAs that could have hinted at the presence of cryptic promoters. (Additional file 3). Therefore, the lack of correlations of the miRNA expression for at least 4 clusters (except the 13b~2c cluster, see above) is unlikely to be explained by independent transcription of the contained miRNA genes.

### Comparison of clustered miR and miR* expression profiles

**Figure 3:**
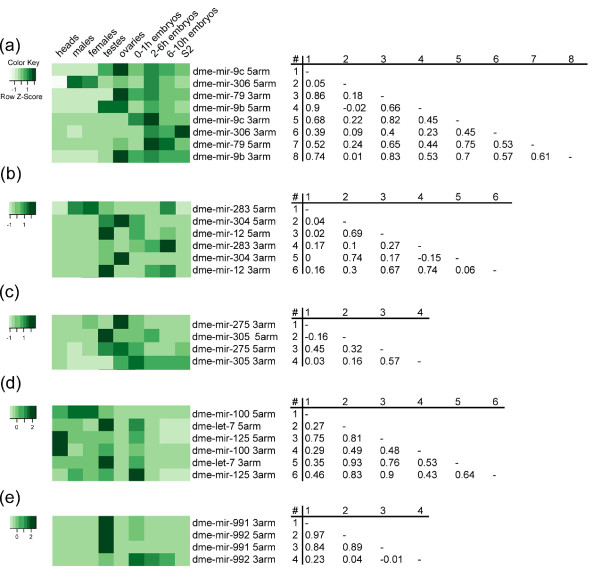
**Expression profiles of clusters containing uncorrelated miRs**. The heatmaps (left) and the correlation tables (right) of miRs and miRs* expression profiles from the 9c~9b **(a)**, the 283~12 **(b)**, the 275~305 **(c)**, the 100~125 **(d) **and the 991~992 **(e) **clusters. Top miRNAs in the heatmaps and the correlation tables are mature miRs, while the bottom ones are star miRs*.

The post-transcriptional regulation of miRNA maturation can be considered as a possible reason of uncoordinated expression of clustered miRs. If alteration of processing or stability happens to miR or its miR*, then their expression profiles may differ, leading to the corresponding low correlation coefficient values. To test this assumption, we determined the correlation coefficients of the expression profiles of clustered miRs and miRs*. Indeed, although expression levels of clustered miRs* are comparable to one another (*P*-value = 0.68, t.test), the correlation coefficient of expression profiles of almost all miR/miR* pairs from each coordinated cluster are high and similar (one exception is the 991~992 cluster, see below), while uncorrelated miRs from each uncoordinated cluster have low correlation coefficients with the corresponding antisense miRs* (Figure 3). For instance, the expression profile of dme-mir-306* has a low correlation coefficient with the dme-mir-306 profile (*r *= 0.09) and correlates well to all other miRs in the 9c~9b cluster (Figure 3a).

Interestingly, the expression profiles of miRs* of uncorrelated miRs in 283~12 and 277~34 clusters differ not only from their miRs but also from profiles of the rest of miRs in the cluster. Dme-mir-283* correlates neither to dme-mir-283 itself (*r *= 0.17) nor to dme-mir-304 (*r *= 0.1) or dme-mir-12 (*r *= 0.27) (Figure 3b). Similarly, expression of dme-mir-305* from the 275~305 cluster does not correlate both to dme-mir-305 (*r *= 0.03) and dme-mir-275 (*r *= 0.16) (Figure 3c). This observation can suggest that hairpin processing regulation or independent regulation of stability of not only miRs but also miRs* in some tissue(s) takes place.

Analysis of the pair correlation coefficients of dme-mir-100, dme-let-7 and dme-mir-125 for the 100~125 cluster demonstrates the complex nature of post-transcriptional regulation (Figure 3d). It is hard to interpret unambiguously the data but it seems that independent regulation of dme-mir-100 and dme-let-7 is occurred. In human, mouse and nematode Lin28 protein binds the conserved pre-let-7, inducing hairpin 3'-end uridylation and its degradation [18-21]. Consistently, we propose that in flies the precursor of dme-let-7 is regulated on a level of hairpin stability because expression profiles of dme-let-7 and dme-let-7* are highly similar (*r *= 0.93) but each of them is slightly different from profiles of dme-mir-100 (*r *= 0.27 and 0.35, respectively) and dme-mir-100* (*r *= 0.29 and 0.53, respectively). A weak correlation of dme-mir-100 and dme-mir-100* (r = 0.29) demonstrates the possibility of dme-mir-100 regulation during its maturation. This is confirmed by finding of the noticeable tissue-specific A-to-I editing of dme-mir-100 in male heads and Kc167 cell line [30]. Dme-mir-125 and dme-mir-125* correlate both to dme-mir-100 and dme-let-7, thus complicating the further analysis.

At the same time, miRs and miRs* from almost all clusters with coordinated miR expression profiles have a high correlation coefficient with each other. The only exception is the 991~992 cluster where dme-mir-992* can be considered as a target of post-transcriptional regulation (Figure 3e). In contrast to dme-mir-991*, the expression profile of dme-mir-992* does not correlate to the profiles of dme-mir-991 and dme-mir-992.

One can speculate that clustered miRNAs with uncoordinated expression profiles are transcribed independently and the absence of upstream TSSs is explained by modest sensitivity of the *McPromoter *program. Since 14 of 24 identified pri-pre-miRNAs [22,23] have one or several TSSs predicted by *McPromoter *within 300 nt range from their 5' ends (data not shown), a sensitivity for prediction of miRNAs promoters is equal to 60%. This is consisted with previously published data [26] and indicates a possible underrepresentation of the used TSS set. But the lack of correlation of miRs and miRs* strongly suggests their post-transcriptional regulation. Selective post-transcriptional regulation of pre-miRNAs from polycistronic primary pri-miRNAs was reported earlier for the human pre-mir-18a from the miR17~92 cluster [31] and for pre-mir-27a from the miR23~24 [32]. Here we predict that miRs and miRs* from at least 5 fly miR clusters (283~12, 275~305, 9c~9b, 100~125, 991~992) undergo post-transcriptional regulation.

It is necessary to note that analyzed small RNA libraries were prepared by three different laboratories. It was shown that sequencing efficiencies of individual miRNAs may differ for libraries prepared by different ways [33]. Thus, difference in miRNA read counts between libraries may be caused by differential cloning efficiencies rather than reflecting different expression levels. All libraries used in the analysis were prepared according to the same protocol from Pfeffer et al [34] with some lab-specific variations. The main source of small RNA bias in the read frequencies are 5' and 3' adapter ligation steps since three 3'-end nucleotides of acceptor and one 5' end nucleotide of donor can influence ligation efficiency by T4 RNA ligase 1 [35]. However, the end nucleotide bias for the 3' adapter ligation step can be alleviated by the use of truncated RNA ligase 2, Rnl2(1-249), which has nearly 100% ligation efficiency regardless of the end nucleotides [36]. Since Rnl2 ligase was used by all three laboratories, it is reasonable to assume that cloning biases due to 3' adapter ligation are not significant. Regarding the 5' adapter ligation step performed by T4 RNA ligase 1, three 3'-end nucleotides of 5' adapters (acceptor) are highly efficient and identical (-rArArA) for 7 of 9 libraries, thus this is unlikely to be the source of miRNA cloning bias. Only two libraries were prepared using 5' adapter with different 3'-end nucleotides (-rArUrC) that does not introduce a significant impact on total tissue expression profiles. The subsequent reverse transcription and PCR steps also may have some effect on sequencing efficiencies of miRNAs, but it is determined mainly by primary structure of small RNAs itself but not caused by cloning protocols [35]. Although we cannot completely exclude the possibility of cloning artifacts in some analyzed small RNA libraries altering miRNA expression profiles, we suggest that generally our main conclusions are correct. Expanding the set of tissues by libraries from other sources can increase the number of uncorrelated miRNAs and uncoordinated miRNA clusters as well as change the correlation coefficients of above mentioned miRNAs.

To test our conclusions on the independent data, we have conducted the correlation analysis of miRNA expression profiles with the expanded dataset from the recently published libraries [30]. This expanded dataset contains 53 selected libraries with more than 330 million reads (Additional file 1, Tables S6 and S7). In accord with our observations, the expression profiles of dme-mir-100, dme-mir-992, dme-mir-283 and dme-mir-306 have low correlation coefficients with profiles of the corresponding miRNAs*. The expression profiles of dme-mir-305, dme-mir-100, dme-mir-992 and dme-mir-283 are also weakly correlated to at least one miRNA from their clusters. Thus, although the correlation coefficients for several clustered miRNAs are different, in general the results remain the same.

Several possible mechanisms of miRNA expression regulation have been described, including the modulation of Drosha and Dicer activities, uridylation of pre-miRNAs and miRs, A-to-I editing of pri- and pre-miRNAs (for review see refs. [37,38]). For dme-let-7 and dme-mir-100 the possible mechanisms of their post-transcriptional regulation are the tissue-specific uridylation and A-to-I editing of their precursors respectively (see above). Interestingly, dme-let-7 as well as clustered coordinated dme-mir-960 and dme-mir-983 were also shown to be A-to-I edited in some tissues [30]. However, the expression levels of these miRNAs expression are very low in these tissues, thus explaining an absence of the influence of editing events on their tissue expression profiles. As the rest of uncoordinated clustered miRNAs seem not to undergo editing in the analyzed tissues, the impact of other mechanisms on the regulation of their expression remains to be revealed.

## Conclusions

In this report we demonstrate that although most Drosophila miRNA clusters are co-expressed in the similar set of tissues, the 283~12, 275~305, 9c~9b, 100~125 and 991~992 clusters contain some miRNAs with different tissue expression profiles compared to other miRNAs from the corresponding clusters. This is unlikely to be explained by independent miRNA transcription because clusters have identifiable putative transcription start sites only upstream of their first miRNA. Most probably uncorrelated clustered miRNAs undergo tissue specific post-transcriptional regulation, which is confirmed by a lack of correlation of miR and corresponding miR* expression profiles.

## Methods

### Analysis of small RNA libraries

The following small RNA libraries were obtained from GEO database and used in the analyses: GSM239041 (heads), GSM278695 (males), GSM278706 (females), GSM280085 (testes), GSM280082 (ovaries), GSM286604 (0-1 h embryos), GSM286605 (2-6 h embryos), GSM286607 (6-10 h embryos) and GSM272652 (S2). Reads have been mapped to dm3 genome assembly using megablast software and requiring perfect matching of the first 18 bases. Reads corresponding to annotated miRNA genes were extracted and their counts were used to infer miRNA expression profiles.

### Analysis of expression profiles of miRNAs

To generate heatmaps and to calculate pair Pearson correlation coefficients *r *for miRNA expression profiles, the relative frequencies of the miRs and miRs* abundances from the libraries were used. The Z-score normalization of miRNA expression profiles in heatmaps (rows) was applied. All operations and statistical calculations were performed by scripts in Perl and R languages.

### Prediction of cluster promoters

The TSSs were predicted for the Drosophila genome (rel.5) by the *McPromoter *program [26] and were fetched from the program web-site (http://tools.genome.duke.edu/generegulation/McPromoter006). After mapping the TSSs on the dm3 genome assembly by the UCSC Genome Browser, the positions of cluster TSSs within up to 5 Kb upstream regions were evaluated by the UCSC Table Browser [39,40]. ChIP-seq data on pol II were fetched from modENCODE server http://www.modencode.org and mapped to dm3 using UCSC Genome Browser.

## Authors' contributions

SSR designed the study, carried out the expression and promoter analyses. EB analyzed the small RNA libraries and helped to draft the manuscript. SSR and VAG wrote the manuscript. All authors read and approved the final manuscript.

## Supplementary Material

Additional file 1 **Tables S1, S2, S3, S4, S5, S6 and S7**. Table S1 contains the brief summary of Drosophila miRNA clusters. Table S2 is the list of the analyzed libraries of sequenced small RNAs. Table S3 contains the relative frequencies of miRNAs occurring in the libraries. Table S4 is the list of the putative TSSs of the miRNA clusters within up to 5 Kb upstream regions. Table S5 contains the probabilities to find TSS within inter-miRNA spaces in miRNA clusters. Tables S6 and T7 are the results of the additional analysis of the expanded dataset. Click here for file

Additional file 2 **Heatmaps and correlation tables of clustered miRNA expression profiles**. Click here for file

Additional file 3 **Genetic maps of the miRNA cluster TSSs**. Click here for file
