# Supplementary material for: Evidence for post-transcriptional regulation of clustered microRNAs in Drosophila
Source: BMC Genomics. 2011 Jul 19;12:371. doi: 10.1186/1471-2164-12-371 (PMC3150300; doi:10.1186/1471-2164-12-371)

Additional File 2. The correlation tables and heatmaps of clustered miRNA expression profiles.

To generate correlation tables and heatmaps the tissue expression profiles of mature miRs and star miRs* from Table S3 (Additional File 1) were used. MiRNAs from pre-miRNA arms were considered as miR or miR* if the frequency of former one is higher then the frequency of latter one. In each correlation table miRs are placed in the upper rows and left columns, while miRs* are placed in the bottom rows and right columns. Both heatmaps with (right) and without (left) Z-score normalization are presented. The expression profiles of miRs and miRs* are placed in the upper and bottom half of the heatmaps, respectively.

Cluster 6~309

	mir-6 5arm	mir-5 5arm	mir-4 3arm	mir-286 3arm	mir-3 3arm	mir-309 3arm	mir-6 3arm	mir-5 3arm	mir-4 5arm	mir-286 5arm	mir-3 5arm	mir-309 5arm
mir-6 5arm	-											
mir-5 5arm	0.79	-										
mir-4 3arm	0.79	0.99	-									
mir-286 3arm	0.68	0.91	0.93	-								
mir-3 3arm	0.91	0.94	0.93	0.9	-							
mir-309 3arm	0.95	0.92	0.9	0.82	0.98	-						
mir-6 3arm	0.79	0.6	0.6	0.71	0.83	0.81	-					
mir-5 3arm	0.81	1	0.99	0.93	0.96	0.94	0.66	-				
mir-4 5arm	0.91	0.81	0.8	0.82	0.96	0.96	0.94	0.85	-			
mir-286 5arm	0.82	0.91	0.87	0.66	0.84	0.89	0.46	0.89	0.72	-		
mir-3 5arm	0.99	0.76	0.76	0.62	0.87	0.91	0.73	0.76	0.86	0.81	-	
mir-309 5arm	0.97	0.74	0.73	0.7	0.91	0.93	0.9	0.77	0.95	0.7	0.95	-

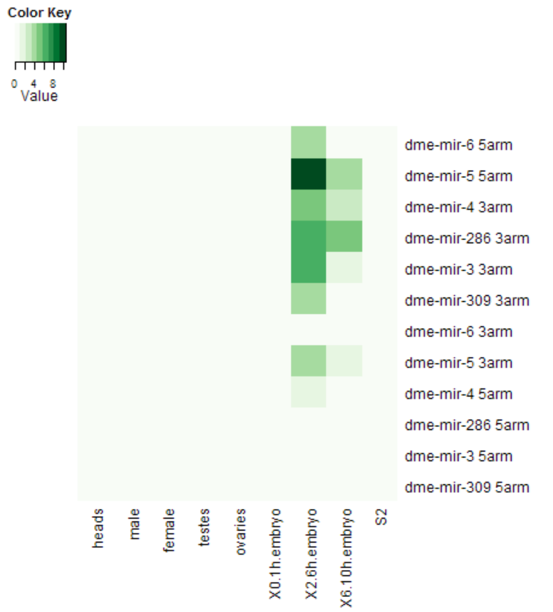

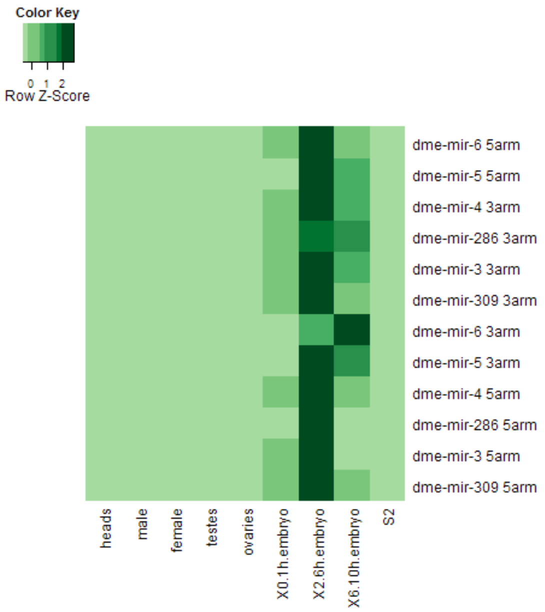

Cluster 100~125

	mir-100 5arm	let-7 5arm	mir-125 5arm	mir-100 3arm	let-7 3arm	mir-125 3arm
mir-100 5arm	-					
let-7 5arm	0.27	-				
mir-125 5arm	0.75	0.81	-			
mir-100 3arm	0.29	0.49	0.48	-		
let-7 3arm	0.35	0.93	0.76	0.53	-	
mir-125 3arm	0.46	0.83	0.9	0.43	0.64	-

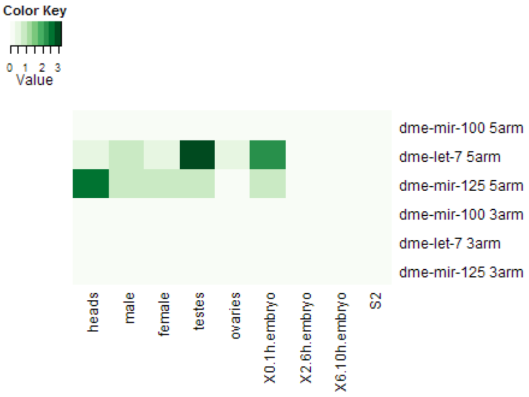

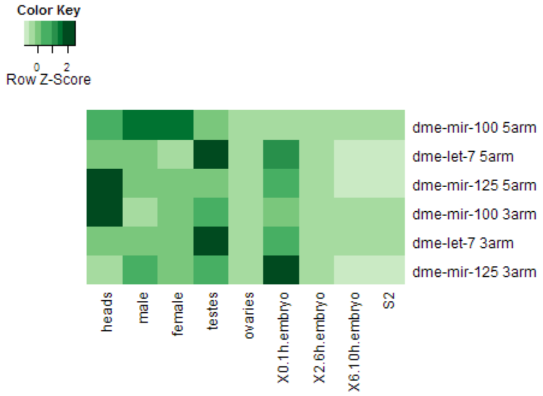

Clusters 310~313 and 991~992

	mir-310 3arm	mir-311 3arm	mir-312 3arm	mir-313 5arm	mir-991 3arm	mir-992 5arm	mir-310 5arm	mir-311 5arm	mir-312 5arm	mir-313 3arm	mir-991 5arm	mir-992 3arm
mir-310 3arm	-											
mir-311 3arm	0.9	-										
mir-312 3arm	0.88	0.83	-									
mir-313 5arm	0.91	0.78	0.96	-								
mir-991 3arm	0.16	0.44	-0.01	-0.13	-							
mir-992 5arm	0.08	0.41	-0.05	-0.19	0.97	-						
mir-310 5arm	0.67	0.6	0.82	0.78	-0.07	-0.11	-					
mir-311 5arm	0.91	0.87	0.94	0.94	0.08	0.04	0.88	-				
mir-312 5arm	0.85	0.85	0.96	0.95	0.01	-0.03	0.76	0.93	-			
mir-313 3arm	0.88	0.91	0.78	0.75	0.5	0.39	0.68	0.85	0.78	-		
mir-991 5arm	0.43	0.65	0.25	0.12	0.84	0.89	0.14	0.35	0.21	0.57	-	
mir-992 3arm	0.56	0.52	0.51	0.58	0.23	0.04	0.37	0.52	0.61	0.71	-0.01	-

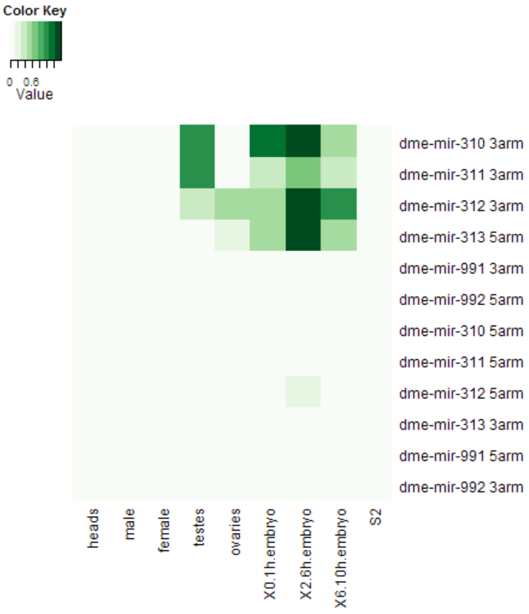

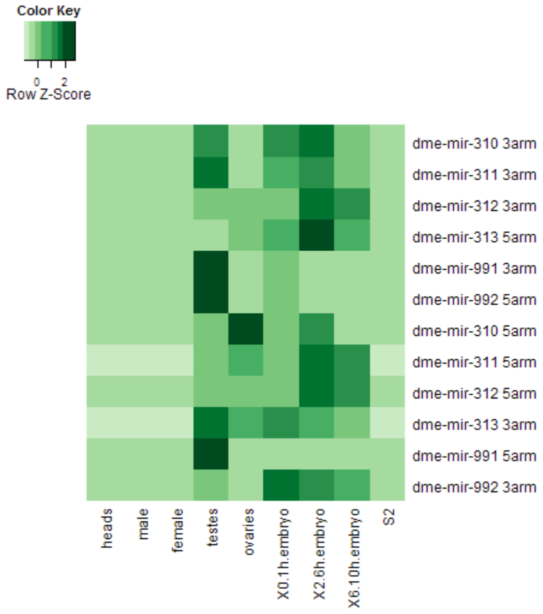

Cluster 982~303

	mir-982 5arm	mir-303 5arm	mir-982 3arm	mir-303 3arm
mir-982 5arm	-			
mir-303 5arm	0.95	-		
mir-982 3arm	0.83	0.66	-	
mir-303 3arm	0.86	0.81	0.89	-

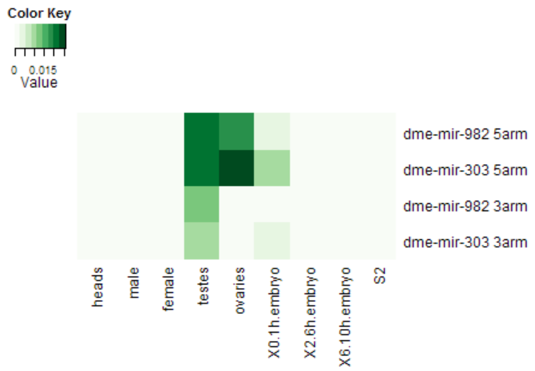

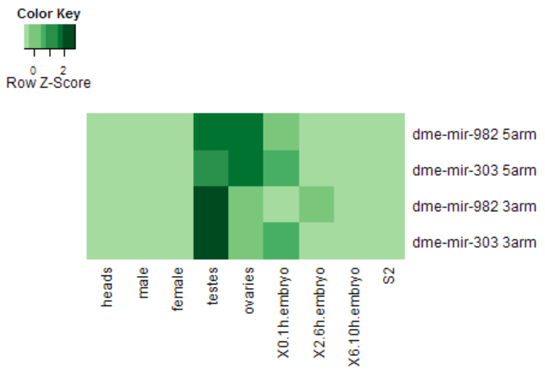

Cluster miRs-959~964

	mir-959 3arm	mir-960 5arm	mir-961 5arm	mir-962 5arm	mir-963 5arm	mir-964 5arm	mir-959 5arm	mir-960 3arm	mir-961 3arm	mir-962 3arm	mir-963 3arm	mir-964 3arm
mir-959 3arm	-											
mir-960 5arm	1	-										
mir-961 5arm	1	1	-									
mir-962 5arm	0.99	0.99	0.99	-								
mir-963 5arm	0.99	0.99	0.99	0.98	-							
mir-964 5arm	1	1	1	0.99	0.99	-						
mir-959 5arm	1	1	1	0.99	0.99	1	-					
mir-960 3arm	1	1	1	0.99	0.99	1	1	-				
mir-961 3arm	1	1	1	0.99	0.99	1	1	1	-			
mir-962 3arm	1	1	1	0.99	1	1	1	1	1	-		
mir-963 3arm	0.95	0.96	0.95	0.95	0.97	0.96	0.95	0.96	0.96	0.96	-	
mir-964 3arm	0.99	0.99	0.99	0.98	1	0.99	0.99	0.99	1	1	0.97	-

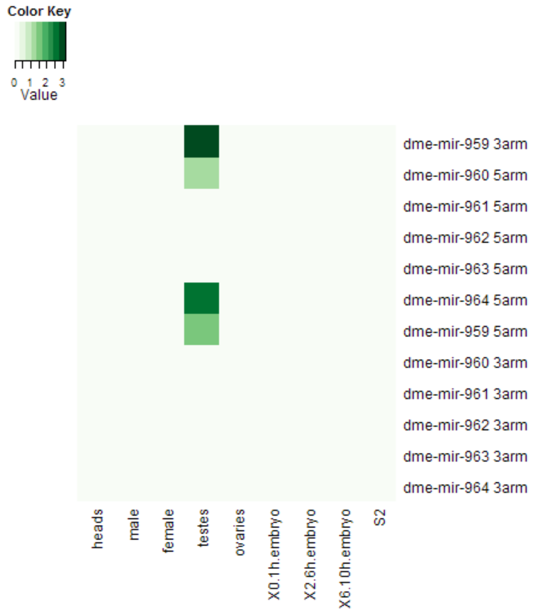

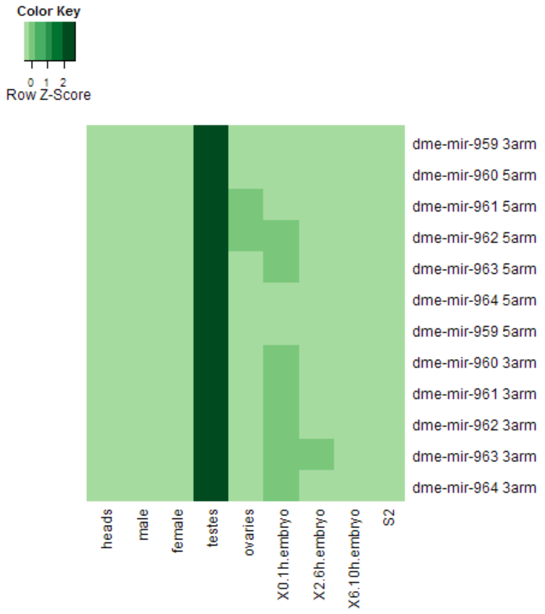

Cluster 978~979

	mir-978 3arm	mir-979 3arm	mir-978 5arm	mir-979 5arm
mir-978 3arm	-			
mir-979 3arm	1	-		
mir-978 5arm	1	1	-	
mir-979 5arm	0.93	0.92	0.93	-

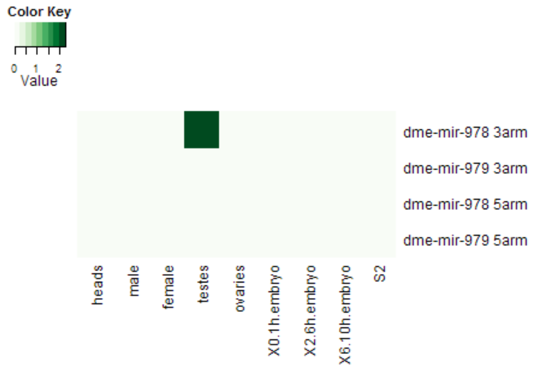

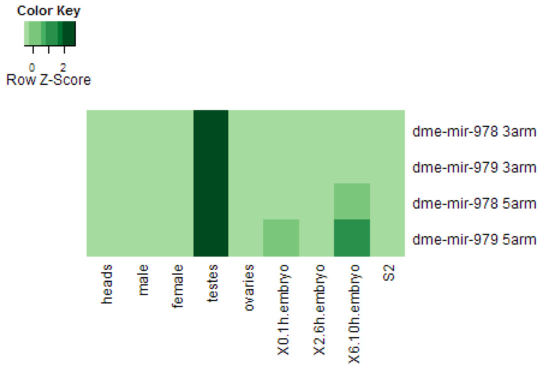

Cluster 972~974

	mir-972 3arm	mir-973 5arm	mir-974 3arm	mir-972 5arm	mir-973 3arm	mir-974 5arm
mir-972 3arm	-					
mir-973 5arm	0.56	-				
mir-974 3arm	1	0.53	-			
mir-972 5arm	0.8	0.65	0.79	-		
mir-973 3arm	0.96	0.58	0.95	0.93	-	
mir-974 5arm	0.99	0.61	0.98	0.84	0.96	-

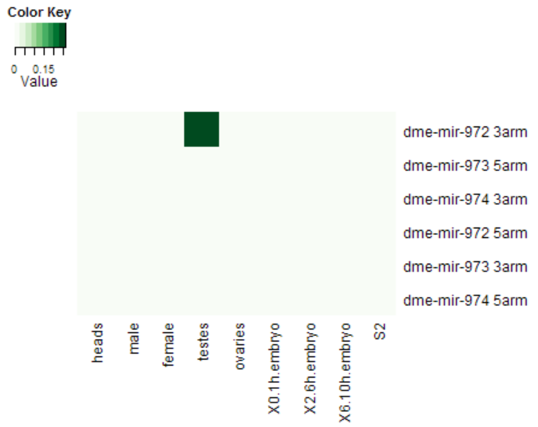

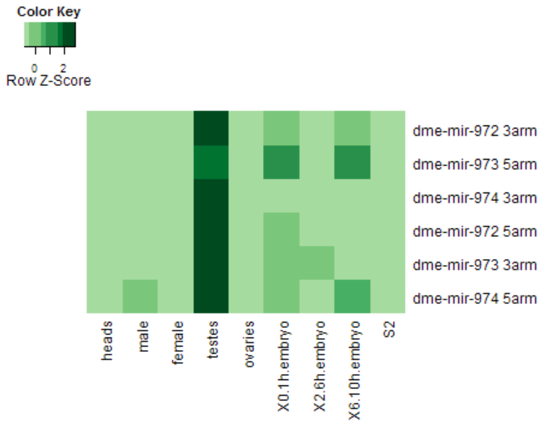

Cluster 975~977

	mir-975 5arm	mir-976 3arm	mir-977 3arm	mir-975 3arm	mir-976 5arm	mir-977 5arm
mir-975 5arm	-					
mir-976 3arm	1	-				
mir-977 3arm	1	1	-			
mir-975 3arm	0.89	0.89	0.88	-		
mir-976 5arm	0.99	0.99	0.98	0.94	-	
mir-977 5arm	1	1	1	0.88	0.99	-

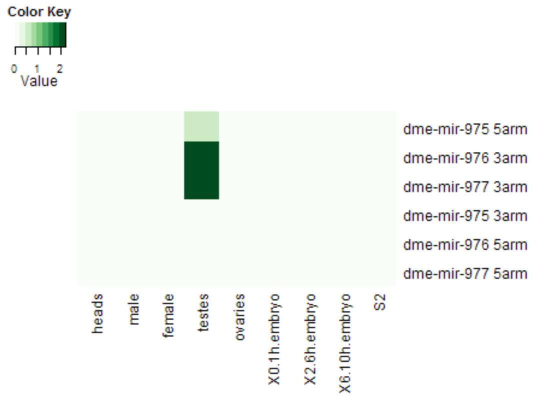

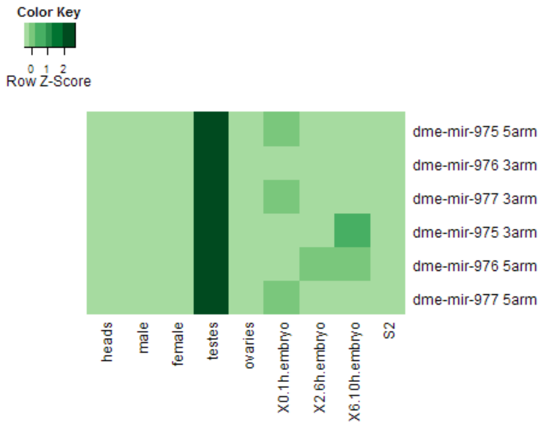

Cluster 1002~968

	mir-1002 5arm	mir-968 3arm	mir-1002 3arm	mir-968 5arm
mir-1002 5arm	-			
mir-968 3arm	0.99	-		
mir-1002 3arm	1	1	-	
mir-968 5arm	0.98	1	0.99	-

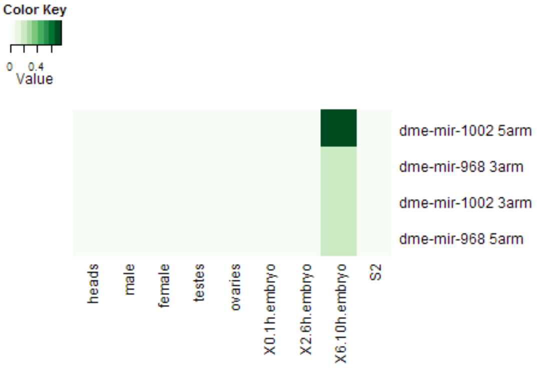

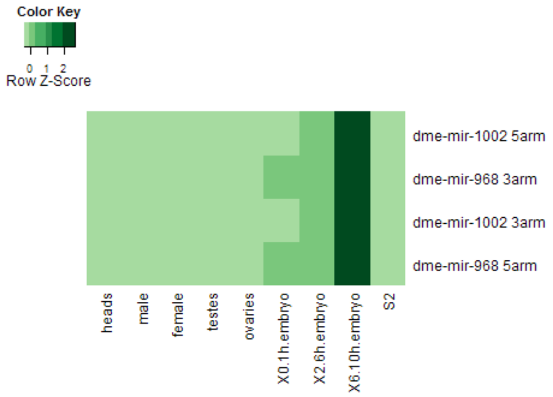

Cluster 2a~2b

	mir-2a 3arm	mir-2b 3arm	mir-2a 5arm	mir-2b 5arm
mir-2a 3arm	-			
mir-2b 3arm	0.86	-		
mir-2a 5arm	0.53	0.58	-	
mir-2b 5arm	0.57	0.63	0.86	-

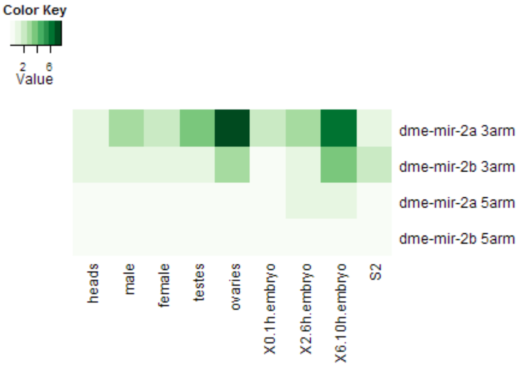

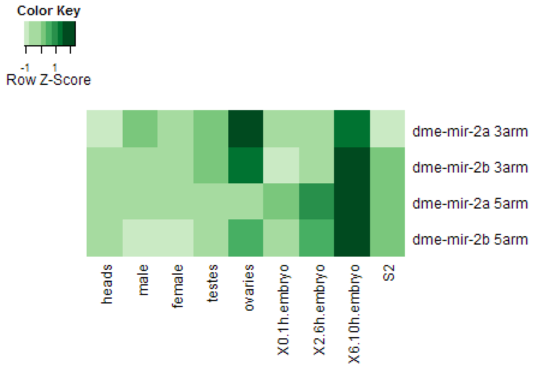

Cluster 275~305

	mir-275 3arm	mir-305 5arm	mir-275 5arm	mir-305 3arm
mir-275 3arm	-			
mir-305 5arm	-0.16	-		
mir-275 5arm	0.45	0.32	-	
mir-305 3arm	0.03	0.16	0.57	-

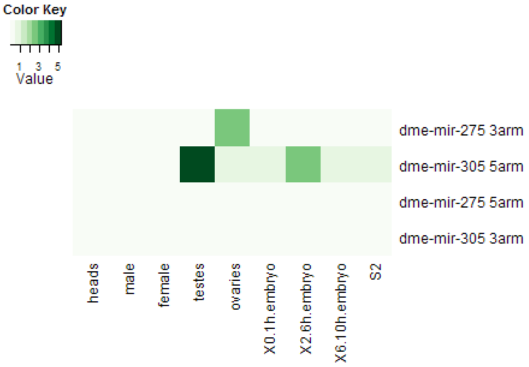

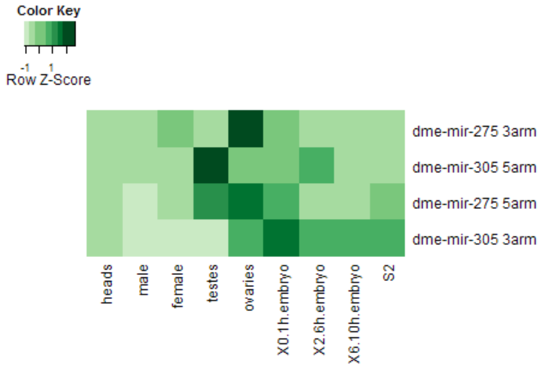

Cluster 9c~9b

	mir-9c 5arm	mir-306 5arm	mir-79 3arm	mir-9b 5arm	mir-9c 3arm	mir-306 3arm	mir-79 5arm	mir-9b 3arm
mir-9c 5arm	-							
mir-306 5arm	0.05	-						
mir-79 3arm	0.86	0.18	-					
mir-9b 5arm	0.9	-0.02	0.66	-				
mir-9c 3arm	0.68	0.22	0.82	0.45	-			
mir-306 3arm	0.39	0.09	0.4	0.23	0.45	-		
mir-79 5arm	0.52	0.24	0.65	0.44	0.75	0.53	-	
mir-9b 3arm	0.74	0.01	0.83	0.53	0.7	0.57	0.61	-

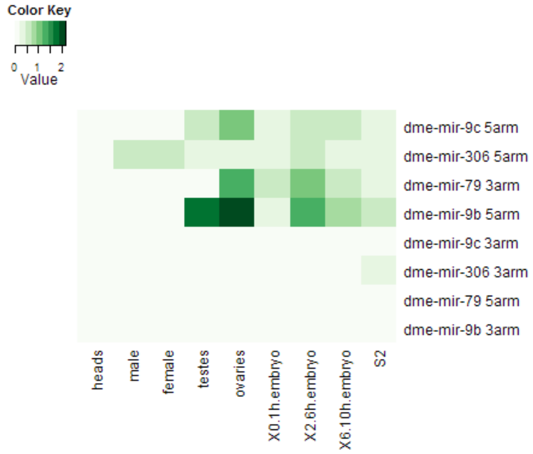

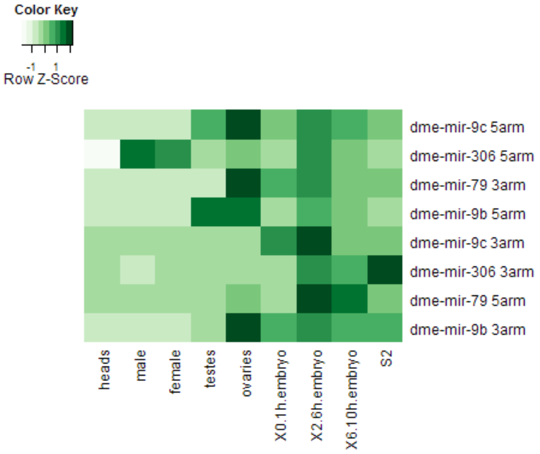

Cluster 998~11

	mir-998 3arm	mir-11 3arm	mir-998 5arm	mir-11 5arm
mir-998 3arm	-			
mir-11 3arm	0.63	-		
mir-998 5arm	0.74	0.56	-	
mir-11 5arm	0.8	0.45	0.92	-

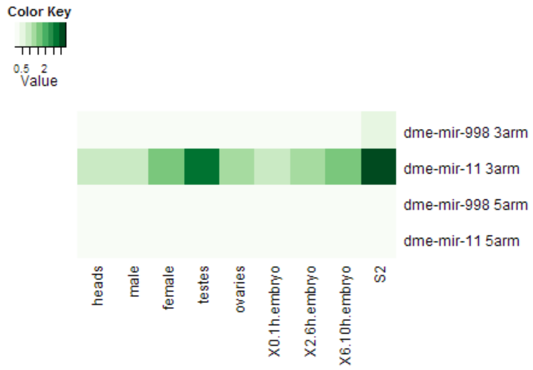

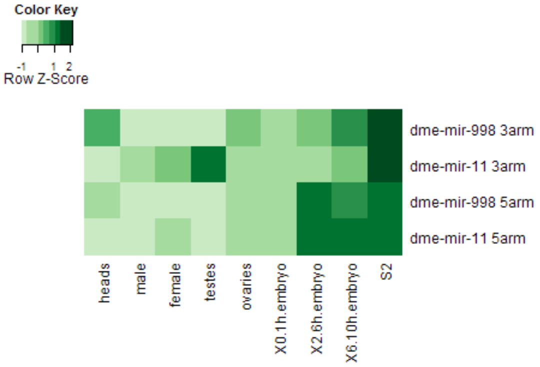

Cluster 277~34

	mir-277 3arm	mir-34 5arm	mir-277 5arm	mir-34 3arm
mir-277 3arm	-			
mir-34 5arm	0.86	-		
mir-277 5arm	0.76	0.95	-	
mir-34 3arm	0.69	0.47	0.47	-

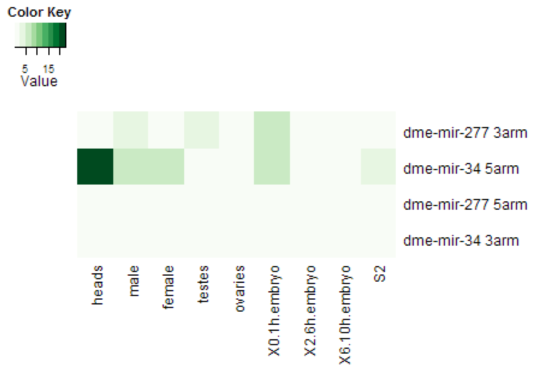

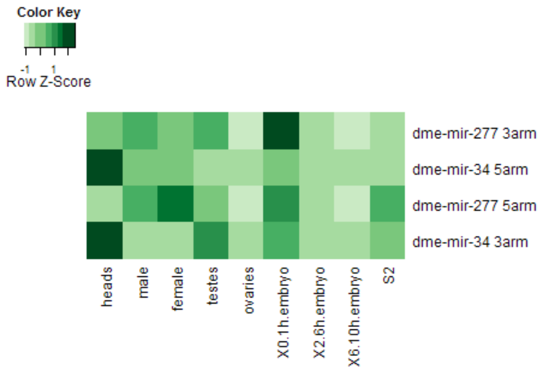

Cluster 994~318

	mir-994 5arm	mir-318 3arm	mir-994 3arm	mir-318 5arm
mir-994 5arm	-			
mir-318 3arm	1	-		
mir-994 3arm	1	1	-	
mir-318 5arm	1	1	1	-

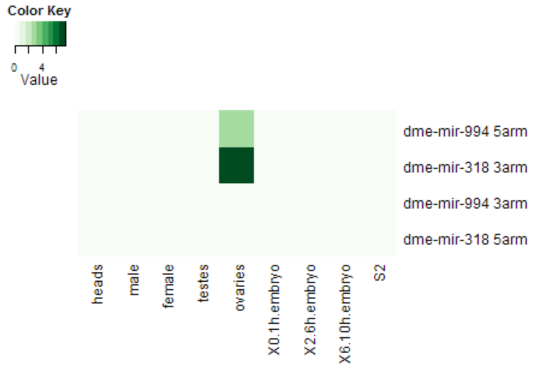

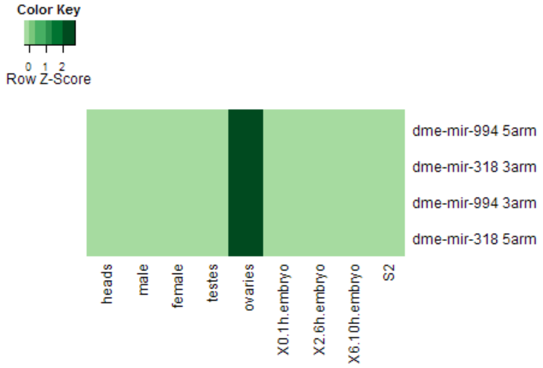

Cluster 983~984

	mir-983 3arm	mir-984 5arm	mir-983 5arm	mir-984 3arm
mir-983 3arm	-			
mir-984 5arm	0.97	-		
mir-983 5arm	0.89	0.97	-	
mir-984 3arm	0.21	0.43	0.63	-

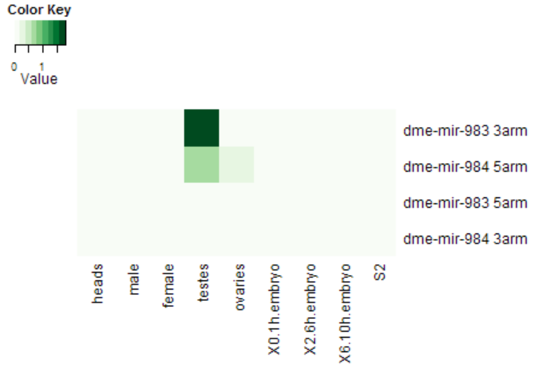

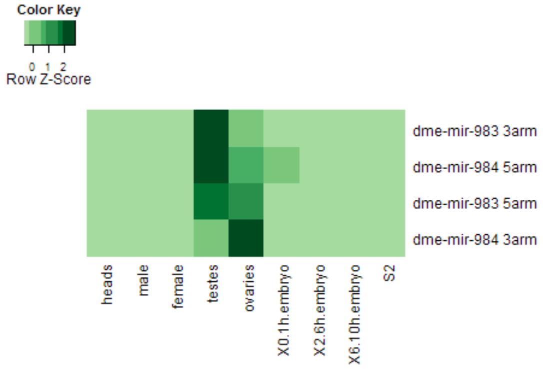

Cluster 283~12

	mir-283 5arm	mir-304 5arm	mir-12 5arm	mir-283 3arm	mir-304 3arm	mir-12 3arm
mir-283 5arm	-					
mir-304 5arm	0.04	-				
mir-12 5arm	0.02	0.69	-			
mir-283 3arm	0.17	0.1	0.27	-		
mir-304 3arm	0	0.74	0.17	-0.15	-	
mir-12 3arm	0.16	0.3	0.67	0.74	0.06	-

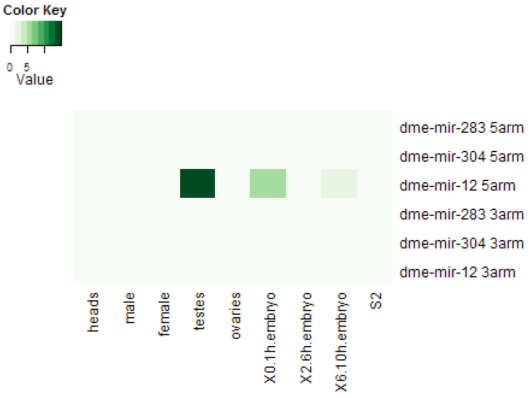

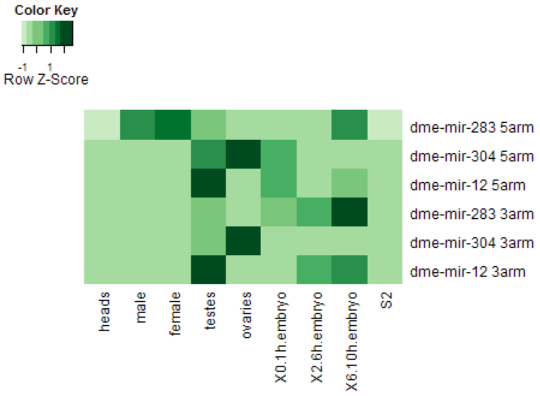

Cluster 13b~2c

	mir-13b 3arm	mir-13a 3arm	mir-2c 3arm	mir-13b 5arm	mir-13a 5arm	mir-2c 5arm
mir-13b 3arm	-					
mir-13a 3arm	-0.21	-				
mir-2c 3arm	0.32	0.34	-			
mir-13b 5arm	0.15	0.11	0.3	-		
mir-13a 5arm	0.4	0.37	0.78	0.64	-	
mir-2c 5arm	0.26	0.41	0.52	0.69	0.9	-

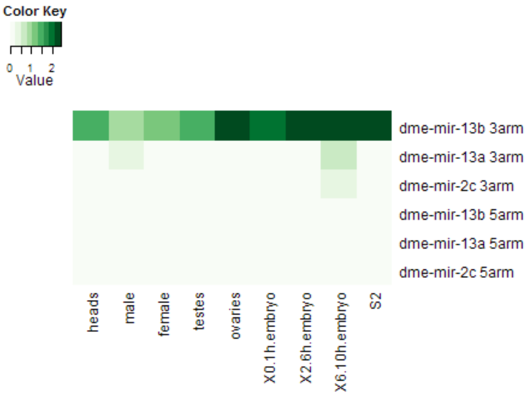

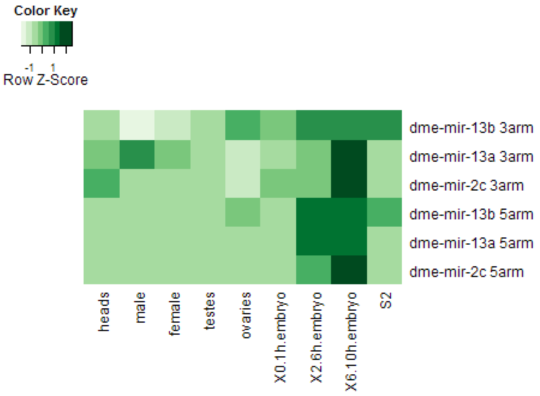

Supplement: Additional file 2 — Heatmaps and correlation tables of clustered miRNA expression profiles. [file 1471-2164-12-371-S2.PDF]
