# Supplementary material for: Evidence for post-transcriptional regulation of clustered microRNAs in Drosophila
Source: BMC Genomics. 2011 Jul 19;12:371. doi: 10.1186/1471-2164-12-371 (PMC3150300; doi:10.1186/1471-2164-12-371)

# Additional File 3. The maps of miRNA cluster TSSs.

Red and blue ticks in TSS track are sense and antisense Transcription Start Sites, respectively.

Cluster 100~125

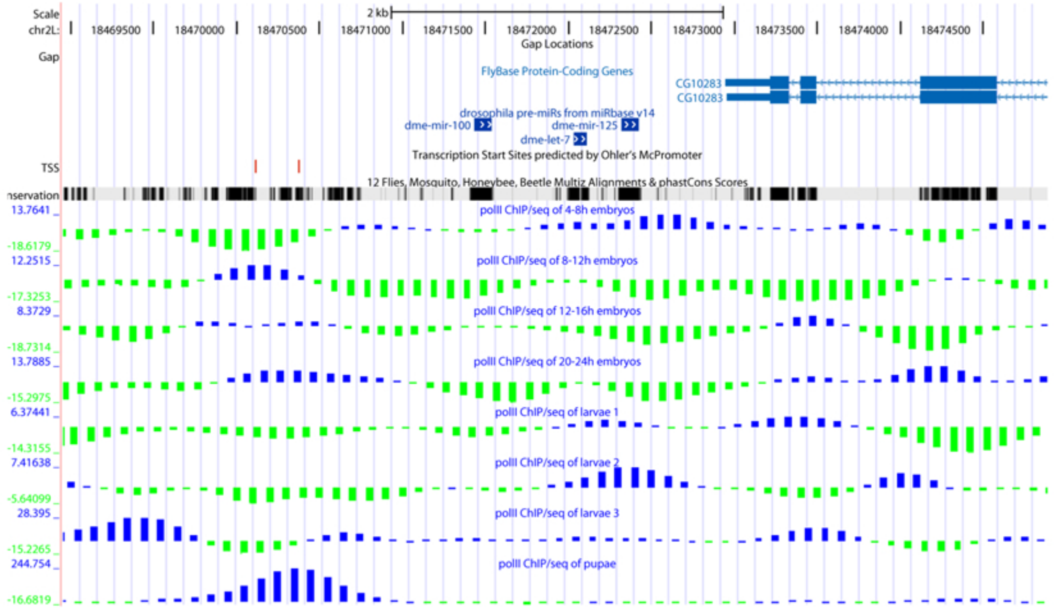


Cluster 1002~968

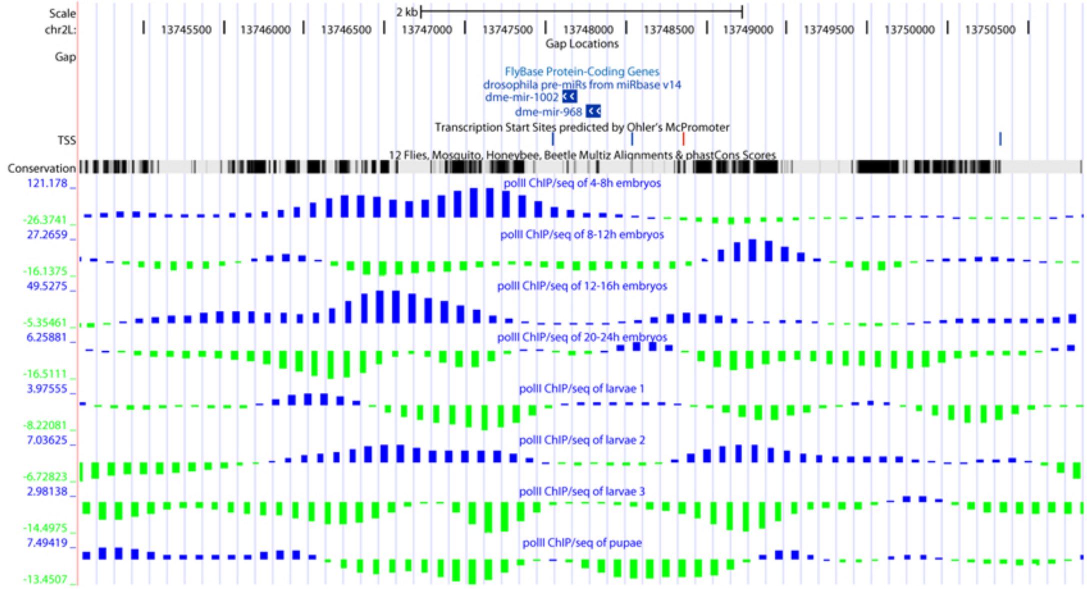


Cluster 13b~2c

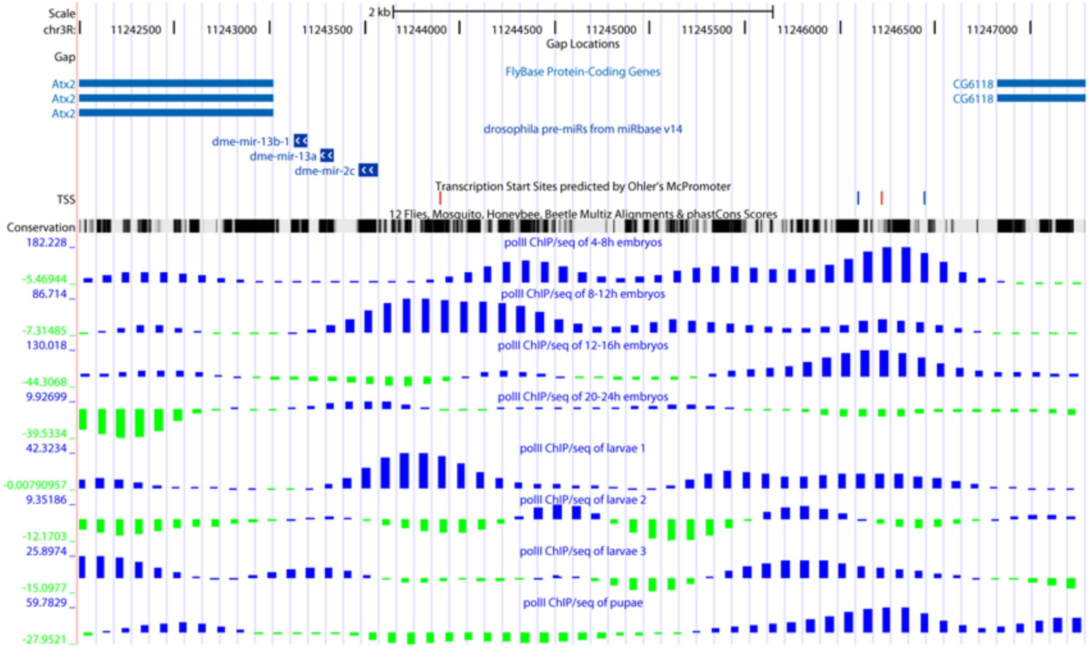

Cluster 2a~2b

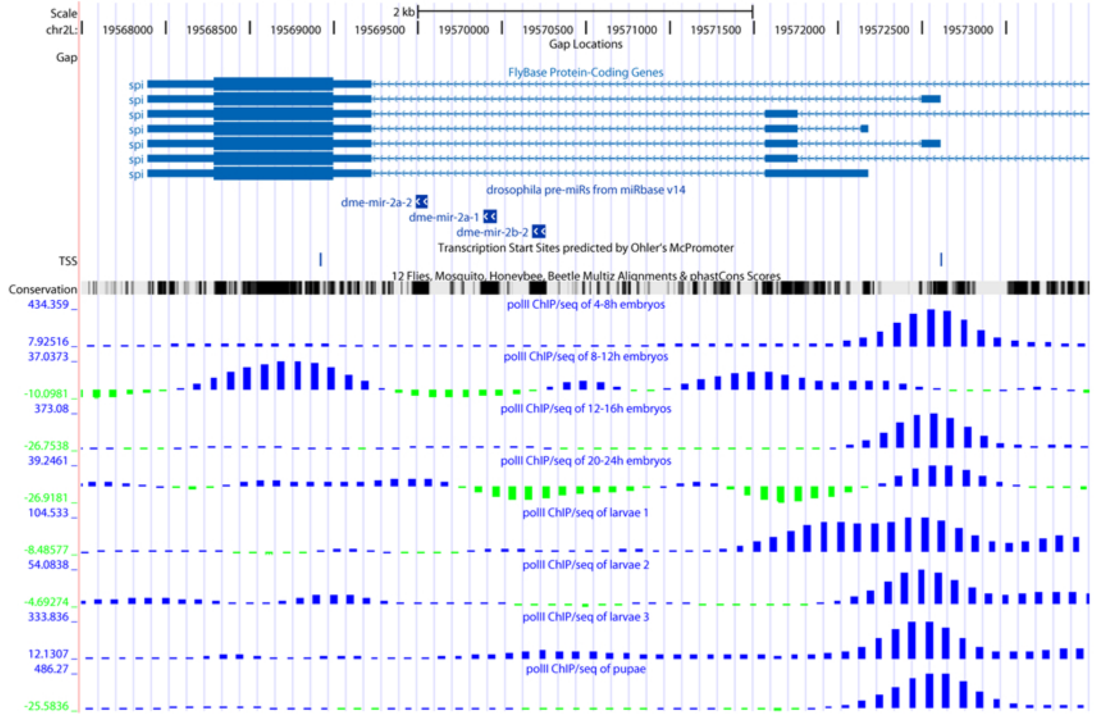


Cluster 275~305

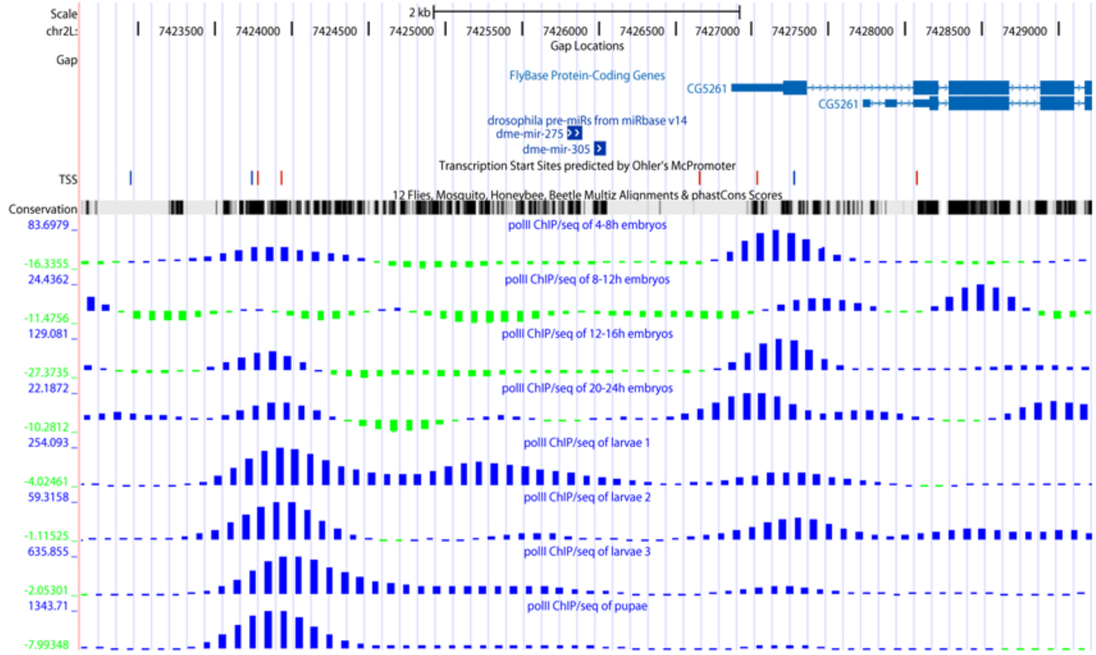


Cluster 283~12

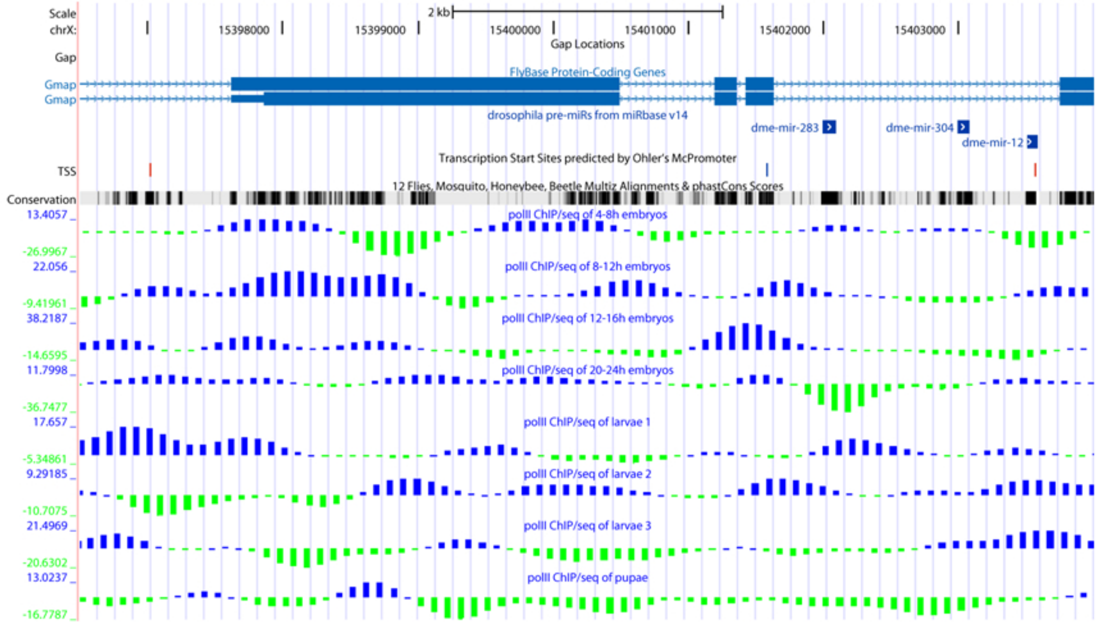

Clusters 310~313
and 911~992

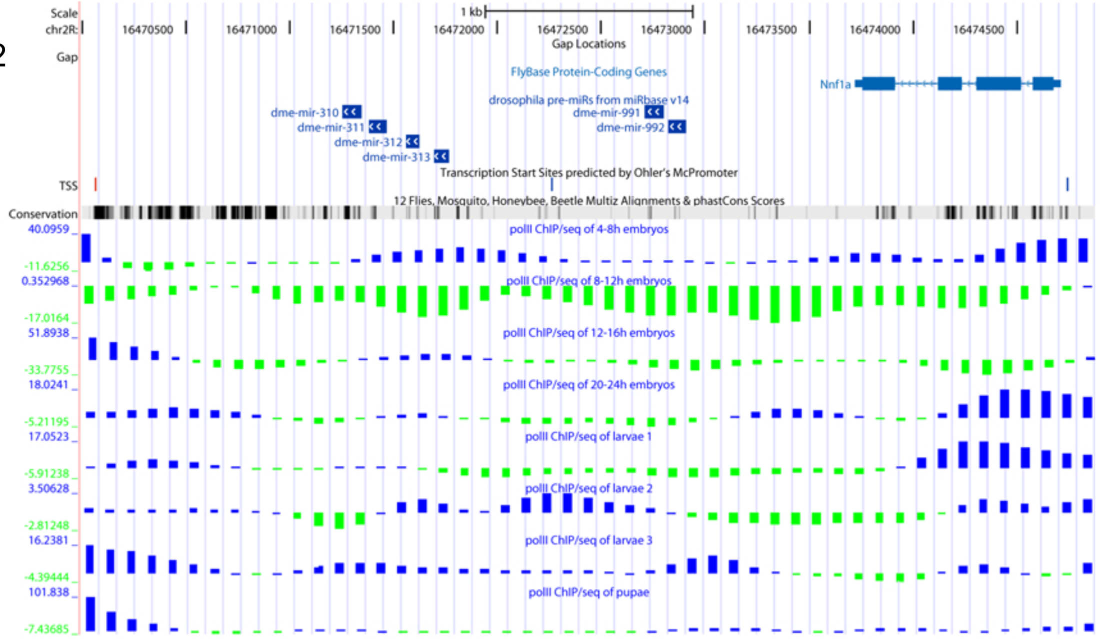


Cluster 6~309

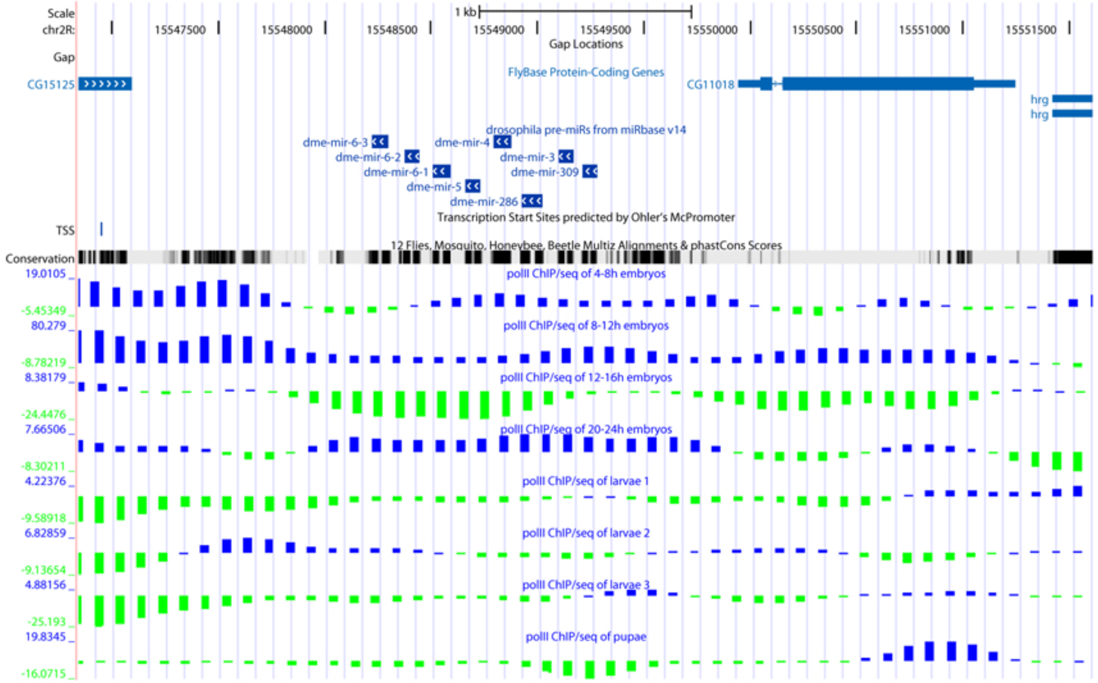


Cluster 9c~9b

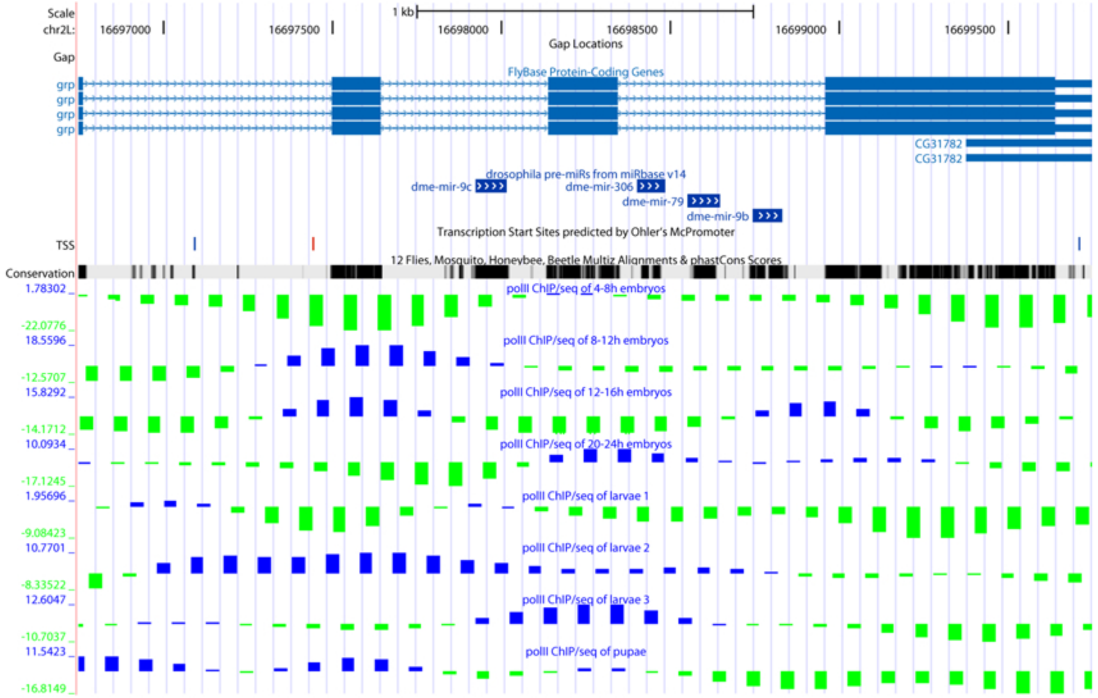

Clusters 959~964

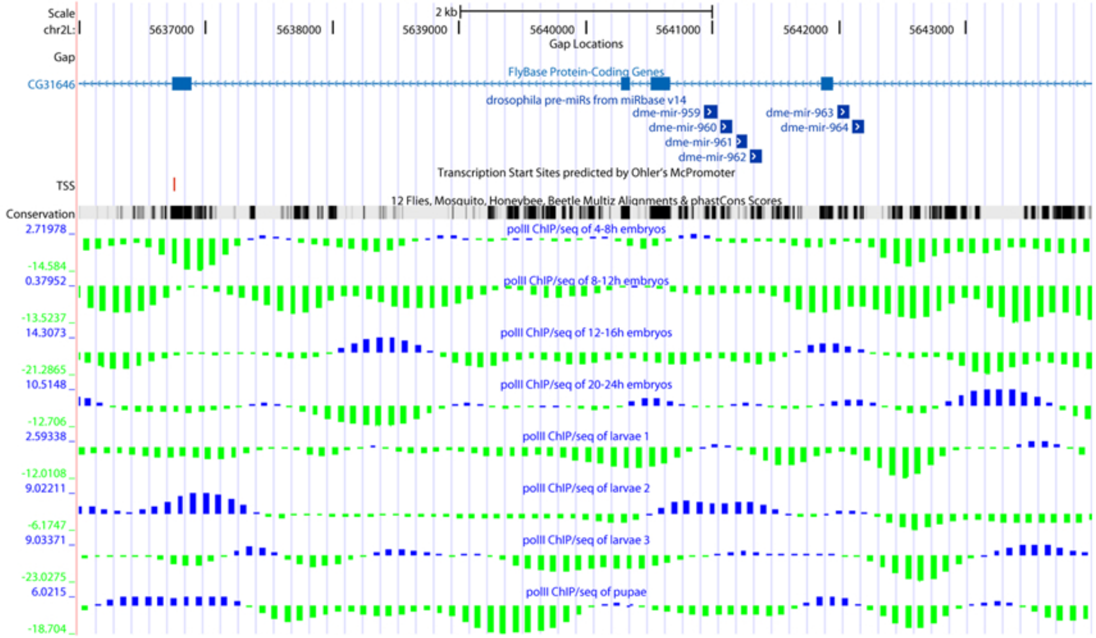


Clusters 972~974,
975~977
and 978~979

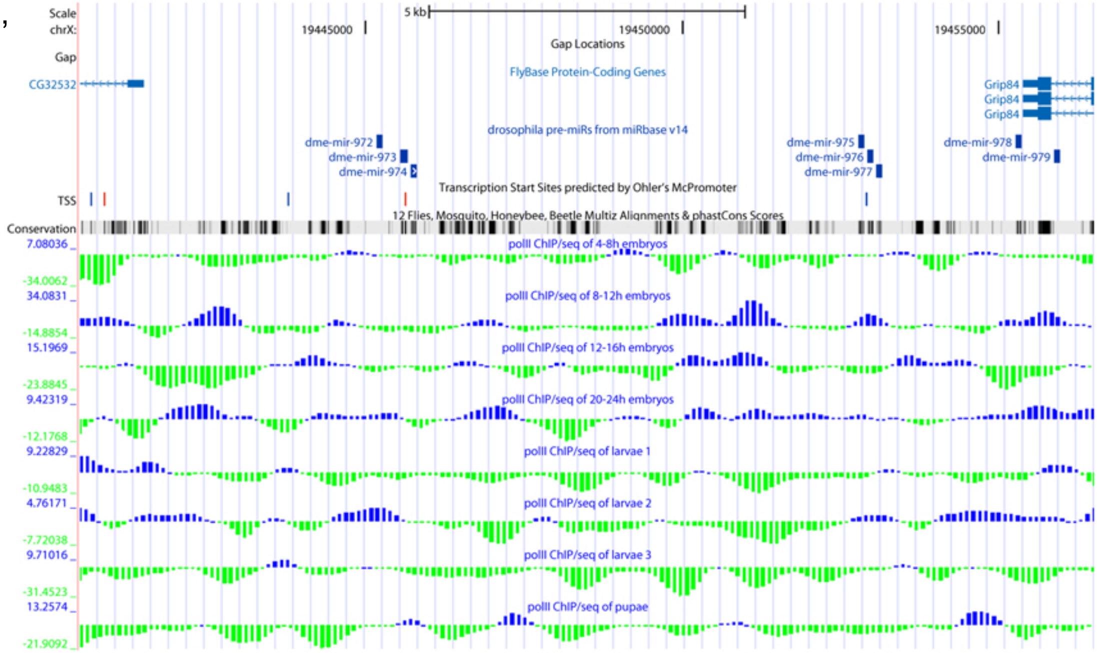


Clusters 982~303
and 983~984

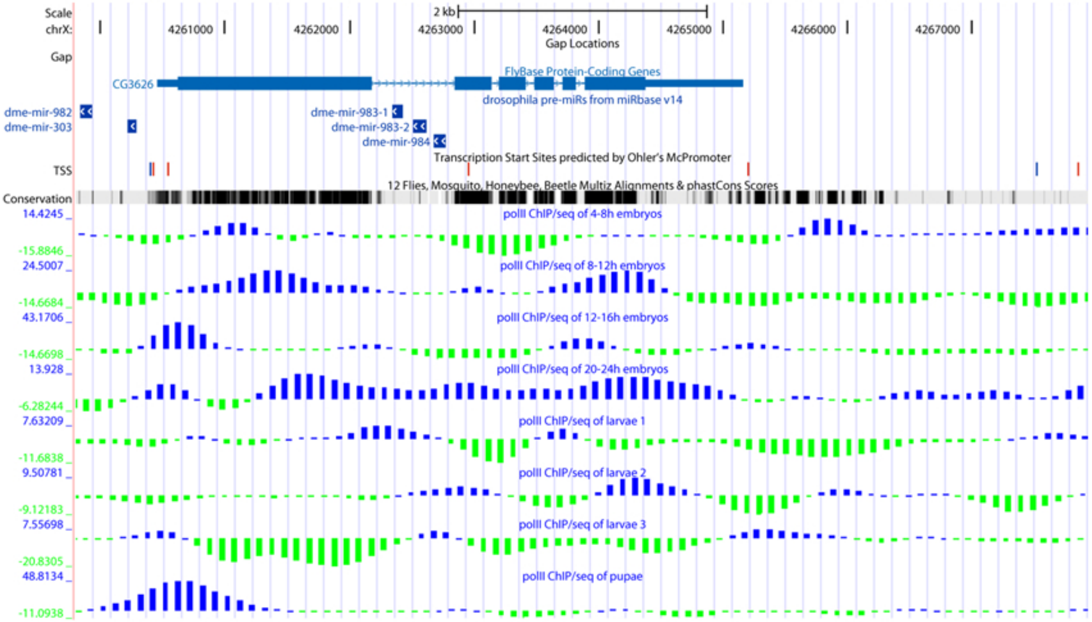

Cluster 994~318

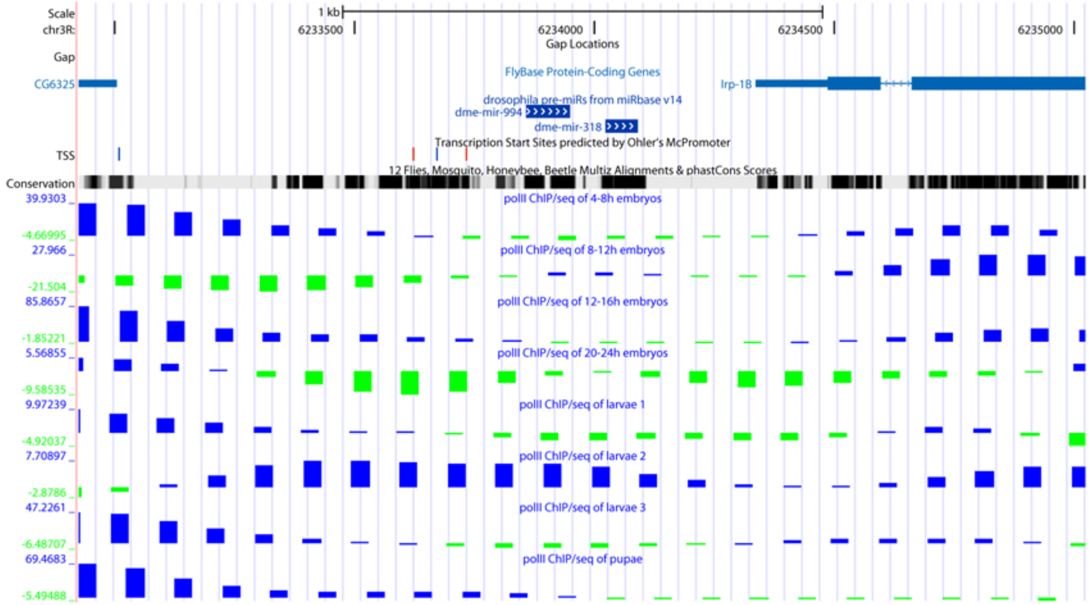


Cluster 277~34

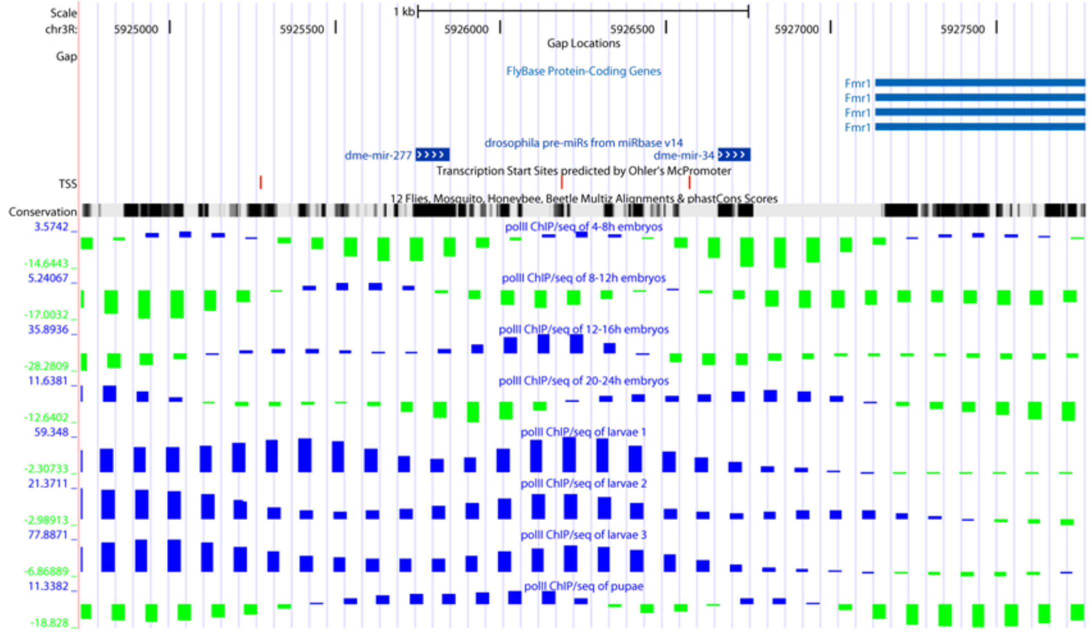


Cluster 998~11

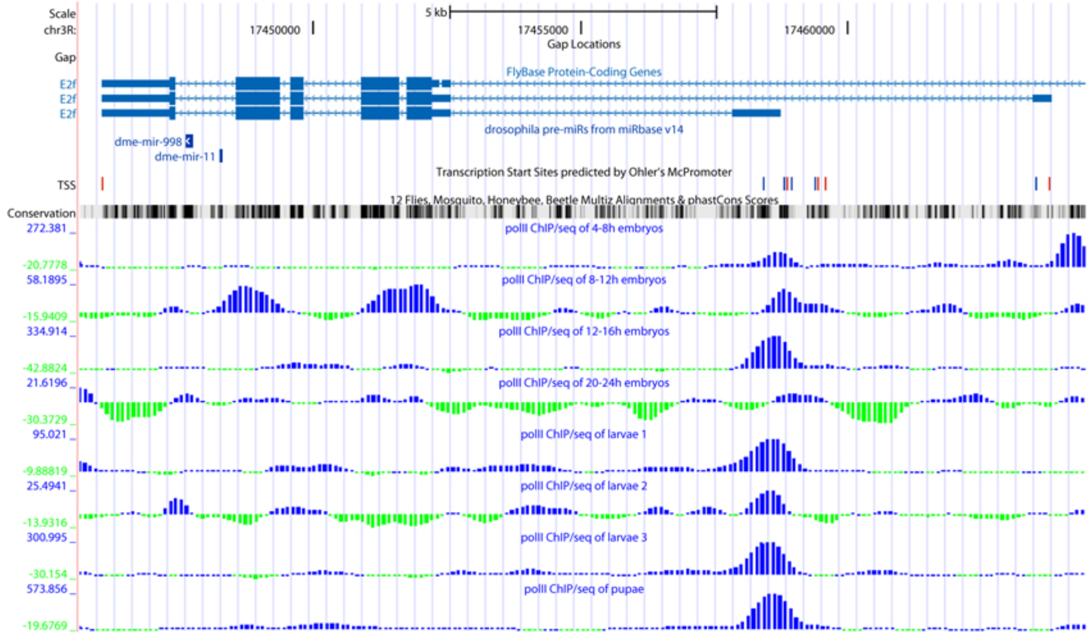

Supplement: Additional file 3 — Genetic maps of the miRNA cluster TSSs. [file 1471-2164-12-371-S3.PDF]
